# WHOOP^H^: whale optimization-based optimal placement of hub node within a WBAN

**DOI:** 10.1038/s41598-024-53889-1

**Published:** 2024-02-10

**Authors:** Shubham Shukla, Vibhav Kumar Sachan, Anurag Sinha, Saroj Kumar Pandey, G. Madhukar Rao, Mohd Asif Shah, Amit Choudhary, Balram Tamrakar

**Affiliations:** 1 Department of Electronics and Communication Engineering, KIET Group of Institutions, Ghaziabad, India; 2 Department of computer science and information technology, IGNOU, New Delhi, India; 3 Department of Computer Engineering and Application, GLA University, Mathura, India; 4 Department of computer science and Engineering, Koneru Lakshmaiah Education Foundation, Hydrabad, India; 5 Kabridahar University, Kabridahar, 250, Somali Ethiopia; 6 Department of Economics, Bakhtar University, Kabul, 2496300 Afghanistan; 7 Centre of Research Impact and Outcome, Chitkara University Institute of Engineering and Technology, Chitkara University, Rajpura, 140401, Punjab India; 8 Chitkara Centre for Research and Development, Chitkara University, Baddi, 174103, Himachal Pradesh India; 9 Department of Electronics and Communication Engineering, Jamia Millia Islamia University, New Delhi, India

**Keywords:** Whale optimization algorithm (WOA), MADM, Wireless body area network, Routing protocol, Clustering, Computational science, Biomedical engineering

## Abstract

Biosensor nodes of a wireless body area network (WBAN) transmit physiological parameter data to a central hub node, spending a substantial portion of their energy. Therefore, it is crucial to determine an optimal location for hub placement to minimize node energy consumption in data transmission. Existing methods determine the optimal hub location by sequentially placing the hub at multiple random locations within the WBAN. Performance measures like link reliability or overall node energy consumption in data transmission are estimated for each hub location. The best-performing location is finally selected for hub placement. Such methods are time-consuming. Moreover, the involvement of other nodes in the process of hub placement results in an undesirable loss of network energy. This paper shows the whale optimization algorithm (WOA)-based hub placement scheme. This scheme directly gives the best location for the hub in the least amount of time and with the least amount of help from other nodes. The presented scheme incorporates a population of candidate solutions called "whale search agents". These agents carry out the iterative steps of encircling the prey (identifying the best candidate solution), bubble-net feeding (exploitation phase), and random prey search (exploration phase). The WOA-based model eventually converges into an optimized solution that determines the optimal location for hub placement. The resultant hub location minimizes the overall amount of energy consumed by the WBAN nodes for data transmission, which ultimately results in an elongated lifespan of WBAN operation. The results show that the proposed WOA-based hub placement scheme outperforms various state-of-the-art related WBAN protocols by achieving a network lifetime of 8937 data transmission rounds with 93.8% network throughput and 9.74 ms network latency.

## Introduction

**Figure 1:**
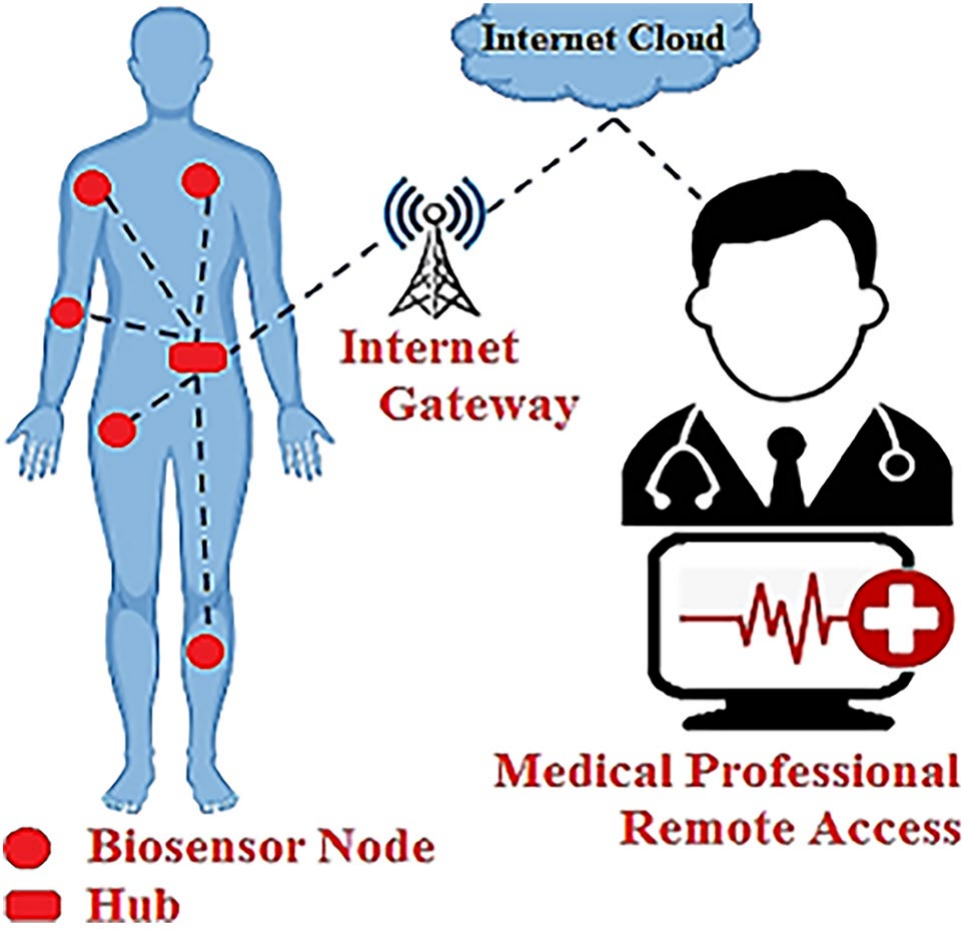
A WBAN system.

A wireless body area network, also known as a WBAN, is comprised of multiple biosensor nodes that are capable of measuring different physiological parameters of the human body^1–4^. Some of these physiological parameters include body temperature, blood pressure, heart rate, etc. The WBAN biosensor nodes are implanted either inside or on the skin of a particular body part as per the measured physiological parameter^5^. The biosensor nodes send physiological parameter information in the form of digital data packets to the hub node^6^. The hub node uploads the physiological data to an internet cloud platform for the purposes of remote health monitoring and telemedicine^7^. A conceptualization of the WBAN system is shown in Fig. 1.

WBAN biosensor nodes are typically built to be smaller in size. Therefore, they contain a small onboard power source; most of that gets exhausted in routing the data packets from source nodes to the hub^1^. Hence, it is important to implement the WBAN system along with an energy-efficient data routing protocol^8,9^. With the help of a data routing protocol, the WBAN selects the optimum data routing pathways. An optimal data routing path offers high-throughput data transmission while consuming minimum node energy^10^. The energy budget-based multi-attribute decision making (EB-MADM) protocol makes use of the hierarchical clustering-based methodology for the purpose of data routing in WBAN^4^. Here, the bio-sensor nodes transmit their data packets to a cluster head (CH) node. The CH performs the process of data aggregation on the received data packets and makes a single packet of them. Further, this combined packet is sent to the hub. The EB-MADM protocol uses the cumulative performance rating-based multi-attribute decision making (CPR-MADM) scheme to select the suitable bio-sensor node as CH. The DSCB^5^, EB-fg-MADM^11^, and MOOF^12^ protocols also incorporate cluster routing in WBANs. Two-hop data transmission along with data aggregation in a clustering approach saves node energy, thereby increasing the network lifetime. The lifetime of a WBAN can further be considerably extended if a data routing protocol is coupled with an effective hub node placement strategy^13^.

A hub node within a WBAN acts as the receiver of the data packets that are transmitted by the source biosensor nodes. Hence, the source node energy consumption in data packet transmission and the network lifetime are substantially impacted by the variations in the location of the hub^14^. In the Sub-Sect. “Problem Statement and Contributions of the proposed work”, a simulation of the network lifetime metric of a WBAN is performed by putting the hub node in four distinct locations on a patient's body. According to the findings, it has been discovered that shifting the position of a hub node can affect the lifetime of a network by up to 28.59%. As a result, the placement of the hub-node in the optimal location becomes a crucial factor in determining the minimized network power consumption of WBAN and its level of effective operation.

In this work, we have proposed a whale optimization algorithm (WOA)-based scheme that is used to place the hub node at an optimized location on the patient’s body for the purpose of achieving minimal consumption of WBAN energy in data routing operations. The whale optimization algorithm (WOA) is a bio-inspired global search optimization heuristic that mimics the foraging behavior of humpback whales to solve diverse linear or nonlinear optimization problems^15,16^. The proposed hub-node placement scheme establishes an initial population of random search agents in the solution space. Each search agent (also called a whale) is comprised of three variables. The first two variables of the whale structure are the tentative “x and y coordinates” of the hub node position within the WBAN while the remaining last variable indicates the ID of the CH node within the WBAN. Therefore, each whale search agent in the WOA population characterizes a random WBAN topology. Thus, each whale search agent in the WOA population provides an arbitrary solution for the problem of hub placement within the WBAN. Further, the proposed WOA-based hub placement scheme estimates the fitness of each whale search agent in its population. The implemented fitness evaluation operator considers the WBAN topology characterized by a particular whale search agent and estimates the amount of energy consumed by the WBAN nodes for data transmission. The estimated amount of WBAN energy consumption is considered to be the fitness of that particular whale search agent. The proposed WOA-based hub placement method converges into an optimal solution that determines the optimal placement of the hub node coupled with an optimal CH node. The resultant optimal topology achieves a minimized consumption of WBAN energy in data routing operations, which ultimately results in an increase in the overall lifespan of the network operation. Moreover, the presented WOA-based hub positioning scheme is integrated with a cluster routing protocol. The proposed method is abbreviated as WHOOP^H^ (whale optimization based optimal placement of hub). According to the findings of the simulated performance results, WHOOP^H^ attains a significant growth in network stability and lifetime periods when compared to existing protocols that are considered to be state-of-the-art. Additionally, the WHOOP^H^ offers improved throughput and latency characteristics.

The following outline constitutes the organizational structure of further portions of the presented paper: “Related works and problem statement” section provides a concise survey of the background literature along with the research challenges, contributions of the proposed work, and an introduction to the Whale Optimization Algorithm. “Models and assumptions” section discusses the different models and assumptions used for the present research work. The proposed WOA-based hub placement scheme is explained in the “Proposed WOA based hub placement scheme” section. “Data routing” section discusses the cluster routing protocol that has been integrated into the proposed hub placement scheme. The evaluation of the performance outcomes can be found in “Protocol simulation” section. The final conclusions on the present work are given in “Conclusions and future scope” section, which also examines the potential research work that could be done in the future.

## Related works and problem statement

This section provides a short overview of relevant state-of-the-art research works along with the problem statement and the contributions that the present study makes. The section ends with an introduction to the whale optimization algorithm.

### Related works

The currently used WBAN topologies implement data routing techniques that are either multi-hop or hierarchical clustering in nature. For instance, the EEPRS^8^ is an example of a multihop protocol that makes use of intermediate relaying nodes. A relay receives the data packet from a source biosensor node and retransmits the received packet to the hub. Relays are selected from the nodes with the greatest residual energy. OCER^6^ is another example of a multihop data routing protocol for WBAN. OCER allocates a cost function (CF) value to each intermediary WBAN node. The cost function value for a node is calculated based on the node’s residual energy, link reliability, and link path loss. The WBAN node possessing the least CF value is chosen as the relay. OCER uniformly distributes the relaying load across the nodes of the network and hence increases the overall lifetime of the network. The CO-LAEEBA protocol^17^ utilizes cooperative multi-hop routing techniques for WBANs. Here, the emergency data is communicated via single-hop data paths, whereas the normal data is communicated using multi-hop data paths. Minimum hop-count data paths, including high-energy intermediary relaying nodes, are chosen for transmission. CO-LAEEBA offers enhanced network lifetime and throughput performance.

The EERP protocol^2^ makes use of the hierarchical clustering-based technique. Here, the biosensor nodes transmit their packets to a node that acts as the cluster head (CH). The CH creates a single data packet by aggregating the packets it receives. This aggregated data packet is subsequently sent to the hub in order to be processed. EERP incorporates a cumulative performance rating-based multi-attribute decision-making (CPR-MADM) technique that evaluates each WBAN node based on different node properties to select an appropriate CH node. Here, a CPR value is estimated for every node, which is the ratio of node energy to the node’s distance to the hub. As a result, the role of cluster head is given to the node that receives the highest CPR value. The EB-MADM^4^ is another hierarchical clustering-based data routing protocol that incorporates the CPR-MADM approach to elect the CH node. Here, the protocol assigns the CPR values to the WBAN nodes, which are a ratio of the node’s residual energy divided by the predicted network energy loss. The WBAN node achieving the highest CPR becomes the CH. The hierarchical clustering-based DSCB protocol^5^ also utilizes the CPR-MADM technique to select an appropriate CH node. Here, the node CPR values are the function of node energy, node transmission power, and the node’s closeness to the hub. The WBAN node achieving the highest composite rating value becomes the CH. Similarly, the other WBAN protocols, such as IM-SIMPLE^18^, RK-EERP^19^, and MOOF^12^, apply the CPR-MADM-based clustering technique for node data transmission in the WBAN.

### Problem statement and contributions of the proposed work

**Figure 2:**
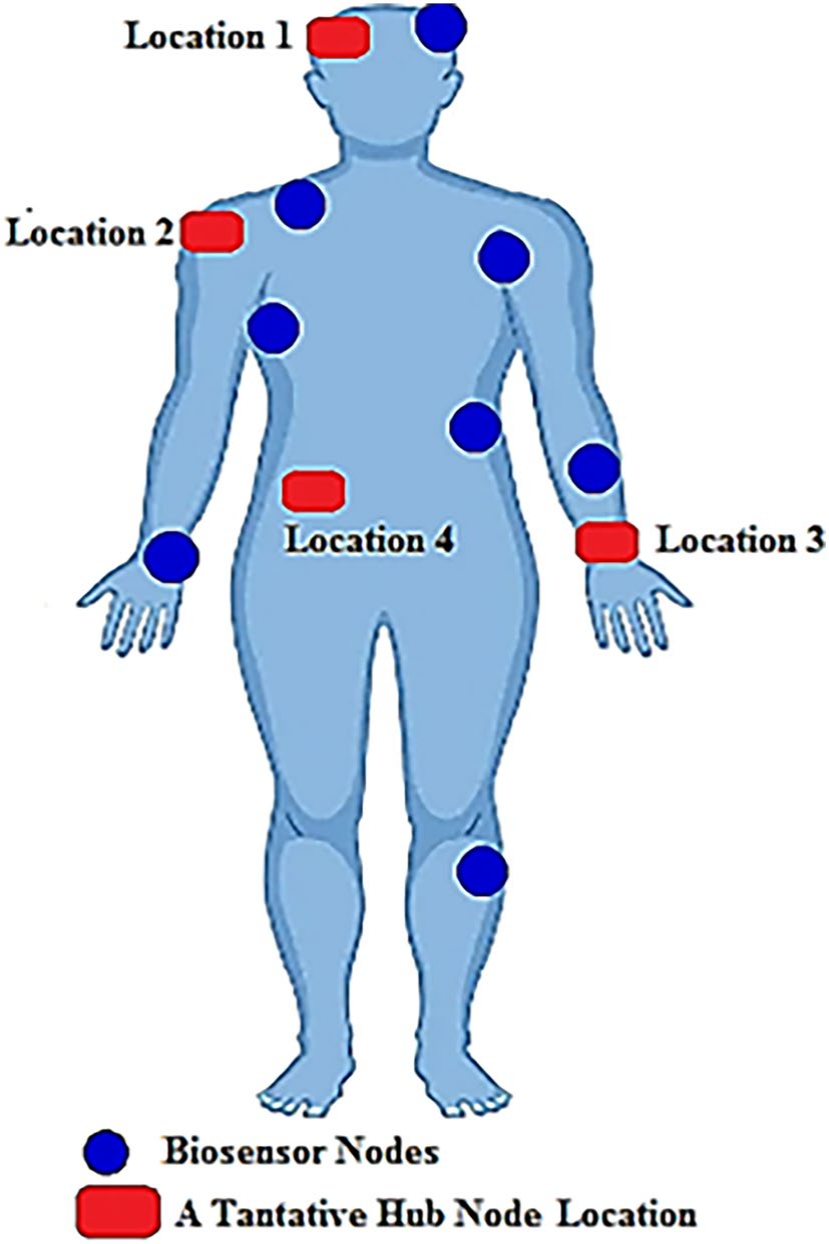
Tantative hub positions with in the WBAN.

**Figure 3:**
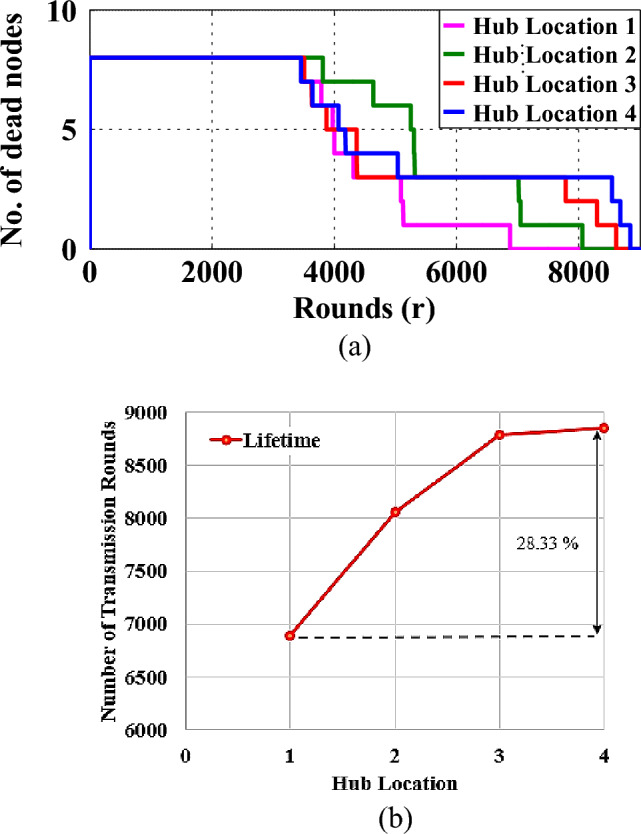
(**a**) Network lifetime for different Hub positions (**b**) Simulated data.

The WBAN data routing protocols discussed in the previous sub-section do not take into account any viable method for the optimal positioning of the hub-node. However, the lifetime of a WBAN is significantly impacted by shifts in the positioning of the hub node^13,14^. In order to demonstrate the precision of this assertion, we carried out a simulation of the lifetime performance of WBAN by positioning the hub node in four distinct positions on a human body during the test. The various hub positions in the WBAN are depicted in Fig. 2. Figure 3a represents the WBAN lifetime performances for various hub node positions whereas Fig. 3b provides an in-depth look at the simulation statistics.

Based on the findings, it has been found that a change in the positioning of the hub node can affect the lifetime of the network by up to 28.59%. Therefore, it is established that the ideal position of the hub node turns out to be a decisive factor that determines the optimal use of node power and the elongated lifespan of WBAN operation. Following are the few studies that have implemented a dedicated technique for deciding the optimum location of the hub node within a WBAN. The link reliability-based hub placement (LRHP) technique was developed by Razavi et al.^20^. As per LRHP, the hub is moved to various body locations, and the reliability of the link between the biosensor nodes and the hub is evaluated in terms of BER. The hub site that provided the lowest BER was chosen as the ideal location for the hub. Yong et al. developed an RSSI-based hub placement (RHP) technique^21^. In this strategy, the hub is repetitively moved to various positions on a human body. Further, the RHP estimates the power of the received signal for every hub location. The hub position offering the highest receiving power readings from WBAN nodes is the ideal position for the hub. Arora et al. developed a reflection coefficient-based hub placement (RCHP) technique^22^. According to RCHP, the reflection coefficient (S11) is measured by putting the hub transceiver antenna at different body locations, and the results are observed. The hub position that recorded the lowest reflection value is chosen as the best possible position for the hub placement.

Huque et al.^13^ termed the hub node as body node coordinator (BNC) and offered three hub node placement techniques. These techniques are referred to as position-aware BNC placement (PBP), distance-aware BNC placement-iterative (DBP-I), and distance-aware BNC placement-fixed (DBP-F). When utilizing these techniques, the hub node is initially positioned outside the WBAN boundary. Further, the hub node performs certain computations based on the relative communication distances of the other nodes and searches for the ideal hub position in an iterative manner.

The hub placement techniques discussed so far are straightforward in terms of computing, but they consume more time as the hub node has to be moved to a variety of locations several times before reaching the optimal location. In addition to this, the participation of the network’s other nodes in the placement process results in an undesirable loss of node energy. Hence, it is important for a hub placement strategy to find an appropriate hub position in a small amount of operational time with minimal participation of WBAN nodes in the hub placement process^13,14^.

Choudhary et al.^14^ utilized particle swarm optimization for hub placement (PSO-HP) in WBAN. The PSO-HP technique requires minimal participation of biosensor nodes during hub placement operations and finds the optimal hub location in the shortest operational time. However, the PSO shows the limitation of falling into local minimum in case of high-dimensional solution space.

The whale optimization algorithm (WOA) implements “Encircling the Prey” and “Bubble Net Feeding” operators through which each whale search agent of the WOA population iteratively updates its position towards the optimal solution. Thus, the WOA improves its solution quality iteratively. Further, the WOA performs the global search by implementing a “random prey search” operator. The random prey search introduces diversity into the WOA population and avoids trapping in the local minimum by preventing the population of whale search agents from becoming too similar to each other. Hence, the WOA discovers the global optimum easily and avoids trapping in the local optimum^15,16^. The PSO does not incorporate a “random search” operator ^23^.The steps of Whale Optimization Algorithm have been discussed in following sub-section. Gou et al.^24^ utilized the Whale Optimization Algorithm for optimal positioning of nodes in general wireless sensor networks (WSNs). However, the Whale Optimization Algorithm has not been utilized for optimal hub node placement in a WBAN system. Table 1 shows the comparison of various hub placement techniques for WBAN.

**Table 1:** Comparison of various hub placement tecnique for WBAN.

Hub placement schemes		Data routing type	Application	Implementation	Accuracy & limitation
Abbreviation	Name				
LRHP^20^	Link reliability-based Hub placement technique	Multi-Hop	Hub placement in WBAN	Evaluation of BER by placing the hub at multiple locations. Best-performing location is selected for hub placement	Limitations: Hub node have to be placed at multiple locations before identifying the optimal hub location Operating procedure is time consuming and requires technical knowledge Involvement of other nodes in the process of hub placement results in an undesirable loss of network energy
RHP^21^	RSSI-based Hub placement technique	Multi-Hop	Hub placement in WBAN	Evaluation of RSSI by placing Hub at Multiple locations. Best-performing location is selected for hub placement	
RCHP^22^	Reflection coefficient-based Hub placement technique	Multi-Hop	Hub placement in WBAN	Evaluation of S_21_by placing Hub at Multiple locations. Best-performing location is selected for hub placement	
PBP^13^	Position-aware BNC placement	Single Hop	Hub placement in WBAN	Hub is placed at multiple locations and node utility is computed based on residual energy and the relative communication distance. Best-performing location is selected for hub placement	Accuracy: Minimal involvement of other nodes in the process of hub placement Limitations: Hub node have to be placed at multiple locations to identifying the optimal hub location Operating procedure is time consuming
DBP-I^13^	Distance-aware BNC placement-iterative	Single Hop	Hub placement in WBAN		
DBP-F^13^	Distance-aware BNC placement-fixed	Single Hop	Hub placement in WBAN		
PSO-HP^14^	Multi-objective optimization framework	Clustering	Hub placement in WBAN	PSO algorithm directly suggest optimal hub coordinates	Accuracy: Operating period is minimal Minimal involvement of other nodes in the process of hub placement Limitations: PSO shows the limitation of falling into local minimum in case of high-dimensional solution space
WHOOP^H^	RK-energy efficient routing protocol	Clustering	Hub placement in WBAN	Whale optimization algorithm directly suggest optimal hub coordinates	Accuracy: Operating period is minimal Minimal involvement of other nodes in the process of hub placement WOA avoids trapping in the local minimum

A whale optimization algorithm (WOA)-based optimal hub node placement strategy (WHOOP^H^) is presented in this paper. The following are the contributions that come with the suggested scheme for the installation of the hub:

- WHOOP^H^ swiftly converges to the optimized solution and directly offers the best location for the hub in the shortest amount of time while still maintaining efficiency.
- WHOOP^H^ does not require the participation of WBAN nodes during the procedure of hub placement.
- WHOOP^H^ is able to explore an expanded solution space in comparison to existing optimization techniques. This capacity allows the process to produce better results.
- WHOOP^H^ is further combined with the CPR-MADM-based cluster-routing protocol to enhance energy efficiency.

According to the findings of our in-depth analysis, the integrated framework of WOA and the CPR-MADM algorithm is being offered as a routing strategy for WBAN systems for the very first time. This marks a significant milestone.

### Whale optimization algorithm

A single objective optimization problem (SOOP) is defined as follows:

- Minimize or maximize *f (ψ)*
- Optimization may or may not be subjected to*A*_*j*_* (ψ)* > *0, j* = *1, 2…J* and *B*_*k*_* (ψ)* = *0, k* = *1, 2…K*

where *f(ψ)* is the objective function that needs to be maximized or minimized; *ψ* is a search agent which is the vector of design variables *ξ*_*i*_; *i*=1,2…*θ* i.e. *ψ*= (*ξ*_*1*_*, ξ*_*2*_*... ξ*_*θ*_); *θ* is the total number of design variables; *β*_*Li*_ is the lower bound and *β*_*Ui*_ is the upper bound of the *ith* design variable *ξ*_*i*_ i.e. *β*_*Li*_≤*ξ*_*i*_ ≤ *β*_*Ui*_.*A*_*j*_* (ψ)* is the *jth* inequality constraint; *J* is the total number of inequality constraint functions;* B*_*k*_* (ψ)* is the *k*^*th*^ equality constraint; *K* is the total number of equality constraint functions.

The whale optimization algorithm (WOA) is a recent bio-inspired global search optimization heuristic that mimics the foraging technique of humpback whales to solve single objective optimization problems (SOOP) i.e. minimize or maximize *f (ψ)*^15^. The foraging behavior of humpback whales includes various maneuvers like bubble-net feeding along with upward-spiral. Here, the humpback whales, after searching for the prey, dive around 12 meters down in the water. After that, they create bubbles in a spiral shape and swim up toward the surface to catch the prey. The WOA performs in the following phases:

#### Whale population initialization

Initially, the WOA generates a population of random search agents *ω*_*P*_= (*ψ*_*1*_*; ψ*_*2*_*; ψ*_*3*_*... ψ*_*η*_) in the solution space. Each search agent is termed the whale. Each whale search agent *ψ*_*i*_; *i*=*1, 2…η* of the WOA population comprises *θ* number of variables i.e. *ψ*_*i*_ = (*ξ*_*i1*_*, ξ*_*i2*_*...ξ*_*ij*_*...ξ*_*iθ*_). The variables *ξ*_*ij*_; *i*=*1, 2…η; j*=*1, 2… θ*of a whale search agent *ψ*_*i*_are initialized using Eq. (1).

1 $${\xi }_{ij}={\beta }_{Lj}+\rho \times \left({\beta }_{Uj}-{\beta }_{Lj}\right) ,$$

where the variable $\rho$ is a random value in between 0 and 1; *β*_*Lj*_ and *β*_*Uj*_are the lower and upper bounds for the variable *ξ*_*ij*_, respectively. The elemental variables of a search agent represent an arbitrary solution to the SOOP under consideration. Hence, a search agent in the whale population is also called the candidate solution.

#### Initial fitness estimation

Further, the WOA estimates the fitness value *F*_*f*_*(i)* of every whale search agent *ψ*_*i*_; *i*=*1, 2…η*of the whale population. WOA uses the objective function *f(ψ)* for fitness estimation. The fitness *F*_*f*_*(i)* of a search agent *ψ*_*i*_ is equal to *f(ψ*_*i*_*)* if the objective function *f(ψ)* is to be maximized. *F*_*f*_*(i)* is equal to 1/*f(ψ*_*i*_*)* if the objective function *f(ψ)* is to be minimized.

#### Calculating the WOA Parameters

After initial fitness evaluation, WOA calculates various parameters such as $a$,$\overrightarrow{A}, \overrightarrow{C,}$ *l* and *p*. Equations (2) to (6) are used for the calculation of WOA parameters.

2 $$a=2-\frac{2*t}{{t}_{max}},$$

3 $$\overrightarrow{A}= 2.a.\overrightarrow{{r}_{1}}-a,$$

4 $$\overrightarrow{C}= 2.\overrightarrow{{r}_{2},}$$

5 $$l={r}_{3},$$

6 $$p= {r}_{4},$$

where the variable *t* denotes the iteration count; the variable *t*_*max*_ denotes the maximum iteration count of the WOA; the vectors $\overrightarrow{{r}_{1}}$ and $\overrightarrow{{r}_{2}}$ denote the random vectors in the range [0, 1]; the variables *r*_*3*_ and *r*_*4*_ are the random numbers in the range [0, 1]. It can be seen that the parameter ‘*a’* is being decreased from the value of 2 to 0 over the course of iterations.

#### Encircling the prey

Further, the WOA identifies the global best solution *ψ*_*best*_ from the initial population. The best candidate solution is the search agent *ψ*_*j*_ that possesses the highest fitness value i.e. *F*_*f*_*(j)*=max [*F*_*f*_*(i)*; *i*=*1, 2…η*].

Now from here onwards, the whale search agents *ψ*_*i*_;*i*=1, 2…η and the global best candidate solution *ψ*_*best*_will be represented as *ψ*_*i*_*(t)* and *ψ*_*best*_*(t)*, respectively where *t*is the WOA-iteration count.

The WOA considers the best search agent as the target prey. Now, if the value of parameters *p*< 0.5 and |A| < 1 then the WOA assumes that the prey is near other search agents and they start updating their positions toward the best candidate solution. This behavior is termed as encircling the prey and is modeled by the Eqs. (7) and (8).

7 $$\overrightarrow{D}= \left|\overrightarrow{C}.{\overrightarrow{\psi }}_{best}\left(t\right)-{\overrightarrow{\psi }}_{i}\left(t\right)\right|,$$

8 $${\overrightarrow{\psi }}_{i}\left(t+1\right)={\overrightarrow{\psi }}_{best}\left(t\right)-\overrightarrow{A}.\overrightarrow{D,}$$

where ${\overrightarrow{\psi }}_{best}\left(t\right)$ is the global best candidate solution i.e. the prey of *t*^*th*^ WOA iteration; ${\overrightarrow{\psi }}_{i}\left(t\right)$ is the *ith* search agent of *t*^*th*^ WOA iteration;${\overrightarrow{\psi }}_{i}\left(t+1\right)$ is the updated *ith* search agent of (*t* + *1)*^*th*^ WOA iteration.

#### Bubble-net feeding method (exploitation phase)

If the value of parameter *p*≥ 0.5 then the WOA implements the bubble-net feeding method of humpback whales along with an upward spiral maneuver. In this case, other search agents update their positions toward the best candidate solution using Eqs. (9) and (10).

9 $$\overrightarrow{{D}{\prime}}= \left|{\overrightarrow{\psi }}_{best}\left(t\right)-{\overrightarrow{\psi }}_{i}\left(t\right)\right|,$$

10 $${\overrightarrow{\psi }}_{i}\left(t+1\right)=\overrightarrow{{D}^{\mathrm{^{\prime}}}}{e}^{bl}{\text{cos}}\left(2\pi l\right)+{\overrightarrow{\psi }}_{best}\left(t\right),$$

#### Random search for prey (exploration phase)

If the value of parameters *p*< 0.5 and |A| ≥ 1 then the WOA assumes that the prey is far away from other search agents and they start searching for the prey, randomly. In this case, other search agents update their positions toward the random solutions using Eqs. (11) and (12).

11 $$\overrightarrow{D}= \left|\overrightarrow{C}.{\overrightarrow{\psi }}_{rand}\left(t\right)-{\overrightarrow{\psi }}_{i}\left(t\right)\right|,$$

12 $${\overrightarrow{\psi }}_{i}\left(t+1\right)={\overrightarrow{\psi }}_{rand}\left(t\right)-\overrightarrow{A}.\overrightarrow{D,}$$

where ${\overrightarrow{\psi }}_{rand}\left(t\right)$ is a random search agent of *t*^*th*^ WOA iteration.

#### Checking boundary conditions

After updating the positions of all search agents of the WOA population, if any variable *ξ*_*ij*_ of any search agent *ψ*_*i*_ violates the boundary conditions (i.e. *ξ*_*ij*_< *β*_*Lj*_ or *ξ*_*ij*_>*β*_*Uj*_) then the value of that variable is made equal to the related lower or upper bound (i.e. *ξ*_*ij*_ = *β*_*Lj*_ if *ξ*_*ij*_< *β*_*Lj*_ and *ξ*_*ij*_ =*β*_*Uj*_ if *ξ*_*ij*_>*β*_*Uj*_).

#### Fitness estimation and updating the best candidate solution

Further, the WOA again assesses the fitness of each whale search agent of the population and updates the best candidate solution i.e. ${\overrightarrow{\psi }}_{best}\left(t+1\right)$.

#### Updating the WOA parameters

Further, the WOA increments the iteration counter by 1 and updates $a$,$\overrightarrow{A}, \overrightarrow{C,}$ *l* and *p* parameters.

**Figure 4:**
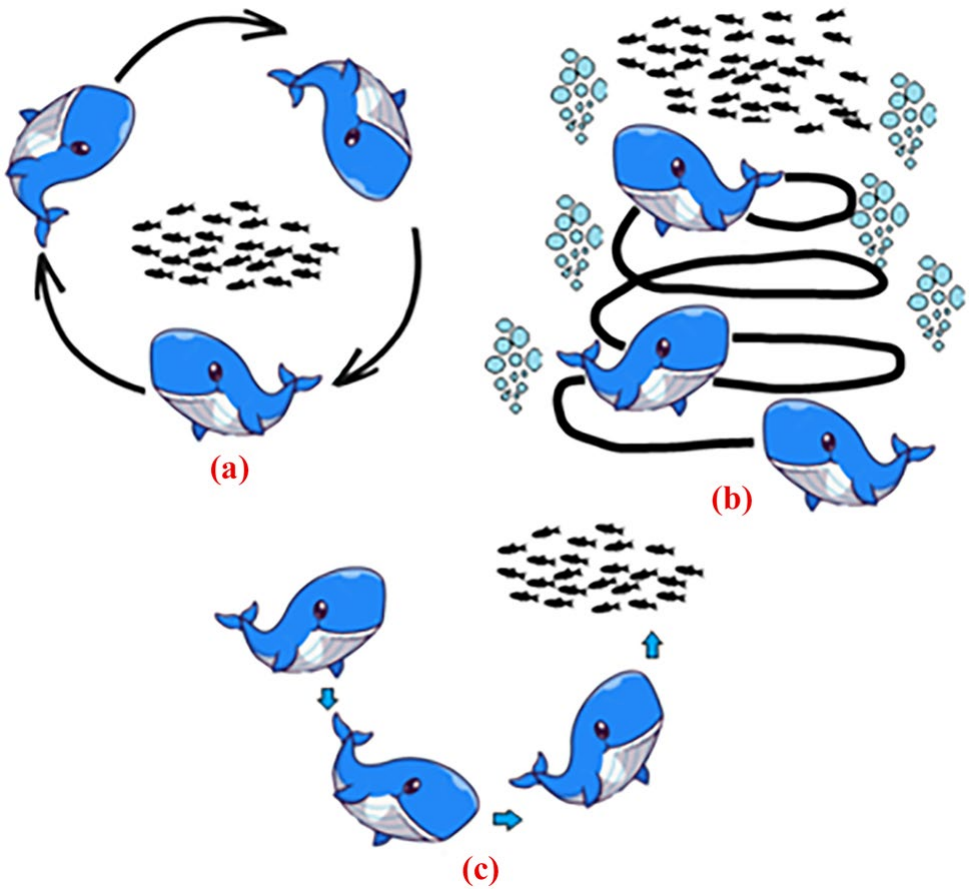
(**a**) Encircling the prey, (**b**) Bubble-net attacking method (exploitation) (**c**) Random search for prey (exploration).

The WOA continues to iterate the steps of Encircling the prey, Bubble-net feeding, Random prey search, Checking boundary conditions, Fitness Estimation & updating the best candidate solution, and Updating the WOA Parameters until the preset iteration count is over. During each iteration, the WOA makes minor adjustments to its whale population to maximize the population's mean fitness. Finally, the WOA develops an optimized whale search agent *ψ*_*opt*_ = (*ξ*_*opt1*_*, ξ*_*opt2*_*... ξ*_*optθ*_) that gives the optimal solution for the target optimization problem. Figure 4a–c show the conceptualization of encircling the prey, bubble-net feeding, and random search for prey maneuvers. Following is a pseudo code of WOA.

**Algorithm 1:**
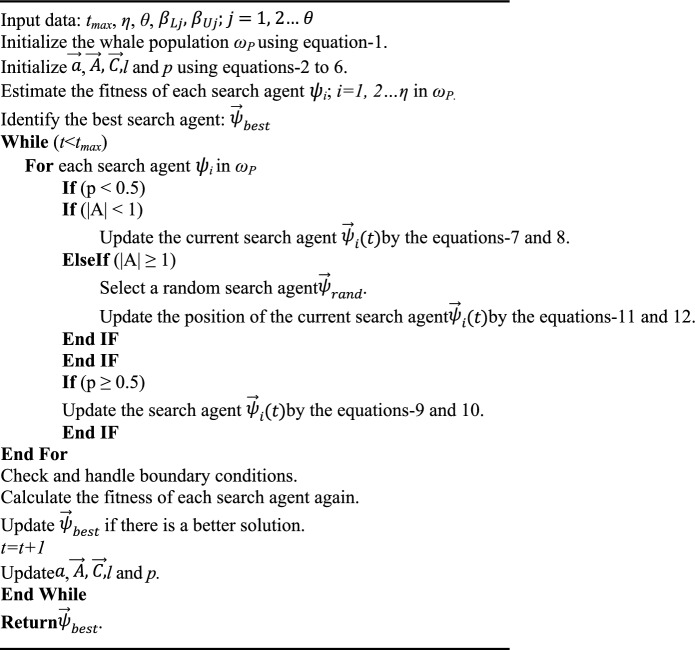
WOA.

## Models and assumptions

The present work uses a WBAN model that comprises eight biosensors for implementing the proposed protocol. Furthermore, the first-order radio energy consumption model^25^ and the Log distance path loss model^6^ are used for protocol simulation. The utilized models are elaborated as follows:

### First-order radio energy consumption model

According to the first order radio energy consumption model, Eqs. (13), (14) and (15) estimate the amount of energy that a node consumes in the transmission, reception and aggregation of a data packet, respectively.

13 $${E}_{T}\left(w,d\right)=\left({E}_{TX}+{E}_{AMP}{ d}^{\gamma }\right)w,$$

14 $${E}_{R}\left(w\right)={E}_{RX}w,$$

15 $${E}_{D}\left(w\right)={E}_{DA}w,$$

where w is the packet’s bit size; *E*_*TX*_ is the energy that a node expends in the transmission of single bit data; *E*_*RX*_ is the energy that a node expends in the reception of single bit data; *E*_*AMP*_ is the energy that a node expends in amplification of single bit data; *E*_*DA*_is the energy that a node expends in the aggregation of single bit data; *d* is the transmission distance; $\gamma$ is the path loss index. Present work overlooks energy expended by the bio-sensor nodes to sense and process the physiological data.

### Path loss model

Equation (16) models the path loss induced to a propagating signal due to the 2.4 GHz on-body communication channel in ISM band.

16 $$pl\left(dB\right)=20.log\left(\frac{4\pi {d}_{o}f}{c}\right)+10\gamma .{\text{log}}\left(\frac{d}{{d}_{o}}\right)+{\chi }_{\sigma }$$

where *f, d*_*o*_*, c,* and ${\chi }_{\sigma }$ refer to the operating frequency (in MHz), the reference distance (in meters), the speed of light (in meters per second), and the shadowing factor of zero mean and $\sigma$ standard deviation, respectively.

### WBAN model

**Figure 5:**
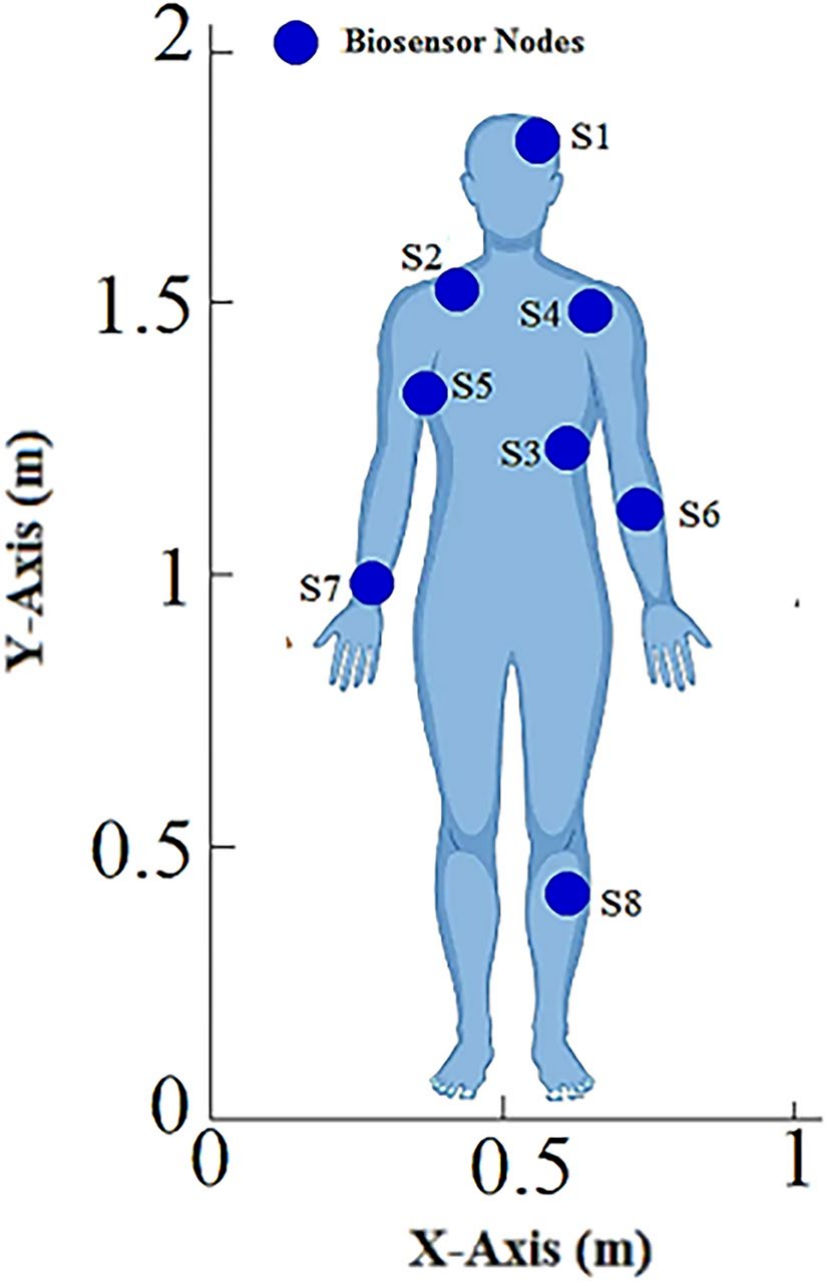
WBAN model.

The work being presented here makes use of a WBAN model that comprises 8 biosensors. The intended WBAN is illustrated in Fig. 5 where Nodes S1 to S6 form a node cluster. The remaining nodes send their data packets to the Hub in a direct connection.

## Proposed WOA based hub placement scheme

The proposed WOA based optimization strategy (WHOOP^H^) is elaborated here as a viable solution for optimal positioning of hub node within the WBAN. The steps involved in the suggested method take place in the sequence as follows:

### Population initialization

**Figure 6:**
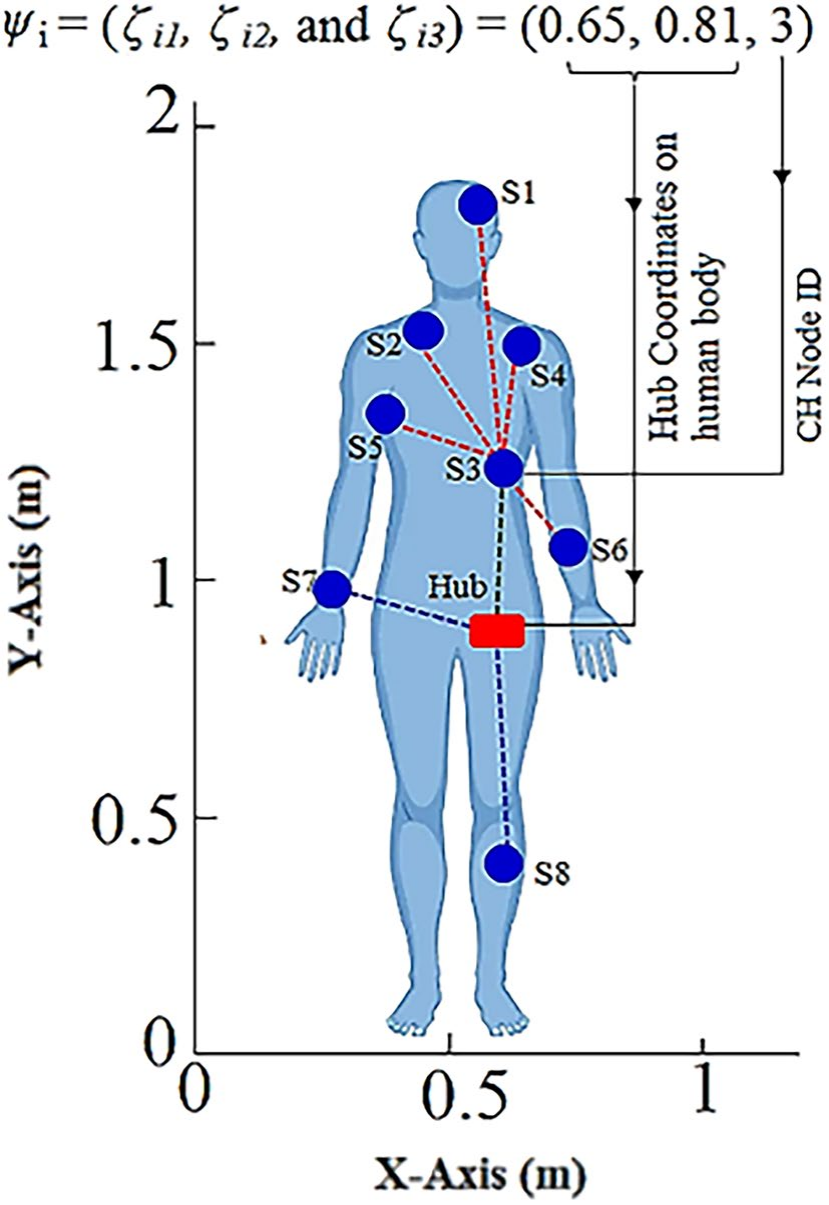
Network topology defined by a whale search agent.

As stated in the “Whale optimization algorithm” sub-section, the WOA first creates an initial population of whale search agents *ω*_*P*_= (*ψ*_*1*_*; ψ*_*2*_*; ψ*_*3*_*... ψ*_*η*_) where the each whale search agent *ψ*_*i*_; *i*=*1, 2…η* includes three variables i.e.*ψ*_*i*_ = (*ξ*_*i1*_*, ξ*_*i2*_*,*and*ξ*_*i3*_). Here, (*ξ*_*i1*_*, ξ*_*i2*_) represents the coordinates of an arbitrary hub node position within the WBAN. Whereas, the third variable*ξ*_*i3*_gives an arbitrary node-ID specifying the CH node in WBAN. The parameter *η* determines the size of the population. The three variables of a whale search agent *ψ*_*i*_ are initialized using Eq. (17).

17 $${\xi }_{ij}=\left\{\begin{array}{c}{\beta }_{Lj}+\rho \times \left({\beta }_{Uj}-{\beta }_{Lj}\right) ;if j=1 or 2\\ {\beta }_{Lj}+Round\left[\rho \times \left({\beta }_{Uj}-{\beta }_{Lj}\right)\right] ;if j=3\end{array},\right.$$

where *β*_*Lj*_ and *β*_*Uj*_are the lower and upper variable bounds, respectively. As a result, each whale search agent in the present population characterizes a random WBAN topology. The WBAN topology as specified by a random whale search agent *ψ*_*i*_ = (*ξ*_*i1*_*, ξ*_*i2*_*,* and *ξ*_*i3*_) = (0.65, 0.81, 3) is shown in Fig. 6.

### Initial fitness evaluation

Next, the WOA estimates the fitness of every whale search agent *ψ*_*i*_; *i*=*1, 2…η*in the form of *E*_*Loss_i*_ which is the amount of energy the network nodes consume for the purpose of sending their data packets to hub node. The WOA considers the network topology characterized by the whale search agent *ψ*_*i*_= (*ξ*_*i1*_*, ξ*_*i2*_*,* and *ξ*_*i3*_) while evaluating its fitness i.e. hub node position at (*ξ*_*i1*_*, ξ*_*i2*_) coordinates along with node id *ξ*_*i3*_ as the CH node. The *E*_*Loss_i*_is given by Eq. (18).

18 $${E}_{Loss\_i}={E}_{L\_non-CH\_i }+{E}_{L\_CH\_i }+{E}_{{L}_{direc{t}_{i}},}$$

The first term“${E}_{L\_non-CH\_i}$” of Eq. (18) represents the amount of energy that the child nodes of the cluster expends in order to communicate their data packets to CH. ${E}_{L\_non-CH\_i}$ is given by Eq. (19) that is derived using first-order radio energy consumption model.

19 $${E}_{L\_non\_CH\_i }=\sum_{\forall j\in U;j\ne {\psi }_{i3}}\left({E}_{TX}+{E}_{AMP}\times {{d}_{j\_CH}}^{\gamma }\right)\times w,$$

where *U* refers to the set of cluster nodes; ${d}_{j\_CH}$ indicates the distance amid node-*j* and the CH that is estimated using Eq. (20).

20 $${d}_{j\_CH}=\sqrt{{\left({X}_{j}-{X}_{{\psi }_{i3}}\right)}^{2}+{\left({Y}_{j}-{Y}_{{\psi }_{i3}}\right)}^{2},}$$

where *(X*_*j*_, *Y*_*j*_*)* denotes the coordinates of node-*j*; (*X*_*ψi3*_*,Y*_*ψi3*_) indicates the coordinates of the CH node.

The second term “${E}_{L\_CH\_i}$”of Eq. (18) represents the amount of energy that the CH node consumesduring data transmission. During a data transmission round, the CH expends its residual energy in following operations:

(i) The CH receives the packets from the child nodes of its cluster. The CH expends ${E}_{RX}(m-1)w\approx {E}_{RX}mw$ energy in receiving the child node packets. Here, *m* represents the total number of nodes included in cluster-*U*.
(ii) Next, the CH carries out the process of data aggregation on child node packets and its own data packet. The CH expands ${E}_{DA} m w$ energy in data aggregation.
(iii) Next, the CH transmits the aggregated data to Hub. The CH expands $\left({E}_{TX}{+E}_{AMP}{{d}_{CH-Hub}}^{\eta }\right)m \delta w$ energy on data transmission. Here, $\delta$ specifies data compression ratio of aggregated data while ${d}_{CH-Hub}$ is the distance in between CH node and the Hub.

Integrating various energy expenditures of CH gives the second term “${E}_{L\_CH\_i}$”. ${E}_{L\_CH\_i}$ is estimated using Eq. (21).

21 $${E}_{L\_CH\_i }={ (E}_{RX}+{E}_{DA}) \times mw+\left({E}_{TX}+{E}_{AMP}{{d}_{CH-Hub}}^{\gamma }\right)\times \delta mw,$$

where the distance ${d}_{CH-Hub}$ is estimated using Eq. (22).

22 $${d}_{CH-Hub}= \sqrt{{\left({X}_{{\psi }_{i3}}-{\psi }_{i1}\right)}^{2}+{\left({Y}_{{\psi }_{i3}}-{\psi }_{i2}\right)}^{2}.}$$

The third term “${E}_{L\_direct\_i}$”of Eq. (18) represents the amount of energy expended by the non-cluster nodes in order to relay emergency data directly to the Hub. Eq. (23) estimates the ${E}_{L\_direct\_i}$.

23 $${E}_{L\_direct\_i }=\sum_{\forall j\in V}\left({E}_{TX}+{E}_{AMP}\times {{d}_{j\_Hub}}^{\gamma }\right)\times w,$$

where *V*indicates the set of nodes that are not part of a cluster; ${d}_{j\_Hub}$ indicates the distance amid non-cluster node-*j* and the Hub. Equation (24) is used to estimate ${d}_{j\_Hub}$.

24 $${d}_{j\_Hub}=\sqrt{{\left({X}_{j}-{\psi }_{i1}\right)}^{2}+{\left({Y}_{j}-{\psi }_{i2}\right)}^{2}.}$$

The fitness of the whale search agent-*ψ*_i_; *i*=*1, 2…η*is given by Eq. (25).

25 $${Fitness}_{i}=\frac{1}{{E}_{Loss\_i}}.$$

### Calculating the WOA parameters

After the initial fitness estimation, the WOA calculates various WOA parameters such as $\overrightarrow{a}$,$\overrightarrow{A}, \overrightarrow{C,}$ *l* and *p*. Equations (2) to (6) are used for calculating the WOA parameters.

### WOA iterative steps

Now the WOA carries out the iterative steps of Encircling the prey, Bubble-net feeding, Random prey search, Checking boundary conditions, Fitness estimation and updating the best candidate solution, and Updating the WOA Parameters. The details of each of the WOA iterative steps is given in “Whale optimization algorithm “sub-section. The WOA, during each iteration, makes minor adjustments to its whale population in order to maximize the mean fitness and minimize the mean amount of energy used by the network. The WOA continues to iterate until the preset iteration count has been reached.

Finally, the WOA develops an optimal solution i.e. the optimized whale search agent *ψ*_*opt*_= (*ξ*_*opt1*_*, ξ*_*opt2*_*,* and *ξ*_*opt3*_) for the placement of the hub node. This ideal solution provides the optimal coordinates for hub placement i.e. (*ξ*_*opt1*_*, ξ*_*opt2*_) and the optimized cluster head i.e. (*ξ*_*opt3*_). The optimal hub coordinates minimize the amount of total energy that is used by the nodes of the network when they transmit their data packets. Hence, the optimal hub coordinates ultimately result in an increase of overall lifespan of the network operation. Thus, the presented (WOA) based hub placement scheme directly gives the optimal hub coordinates and needs minimal involvement of other WBAN nodes in hub placement process.

### Applying the WHOOP^H^ method to WBAN

The suggested WOA based hub placement method (WHOOP^H^) has been applied to the wireless body area network shown in Fig. 5. Table 2 shows various parameters used for WOA based simulation.

**Table 2:** WOA parameters.

Parameter	Value
Number of WOA iterations α	100
Population size *η*	100
Number of variables*θ*	3
Variable lower bounds *β*_*Lj*_	(0.3, 0.5, 1)
Variable upper bounds* β*_*Uj*_	(0.6, 1.5, 6)
*E*_*TX*_	16.7E-09*J/bit*
*E*_*RX*_	36.1E-09 *J/bit*
*E*_*Amp*_	1.97E-09 *J/bit*
*E*_*DA*_	5E-09 *J/bit*
Packet size*w*	4000 bits
Path loss index *γ*	3.38 (LoS)
Data compression ratio $\partial$	0.4

**Figure 7:**
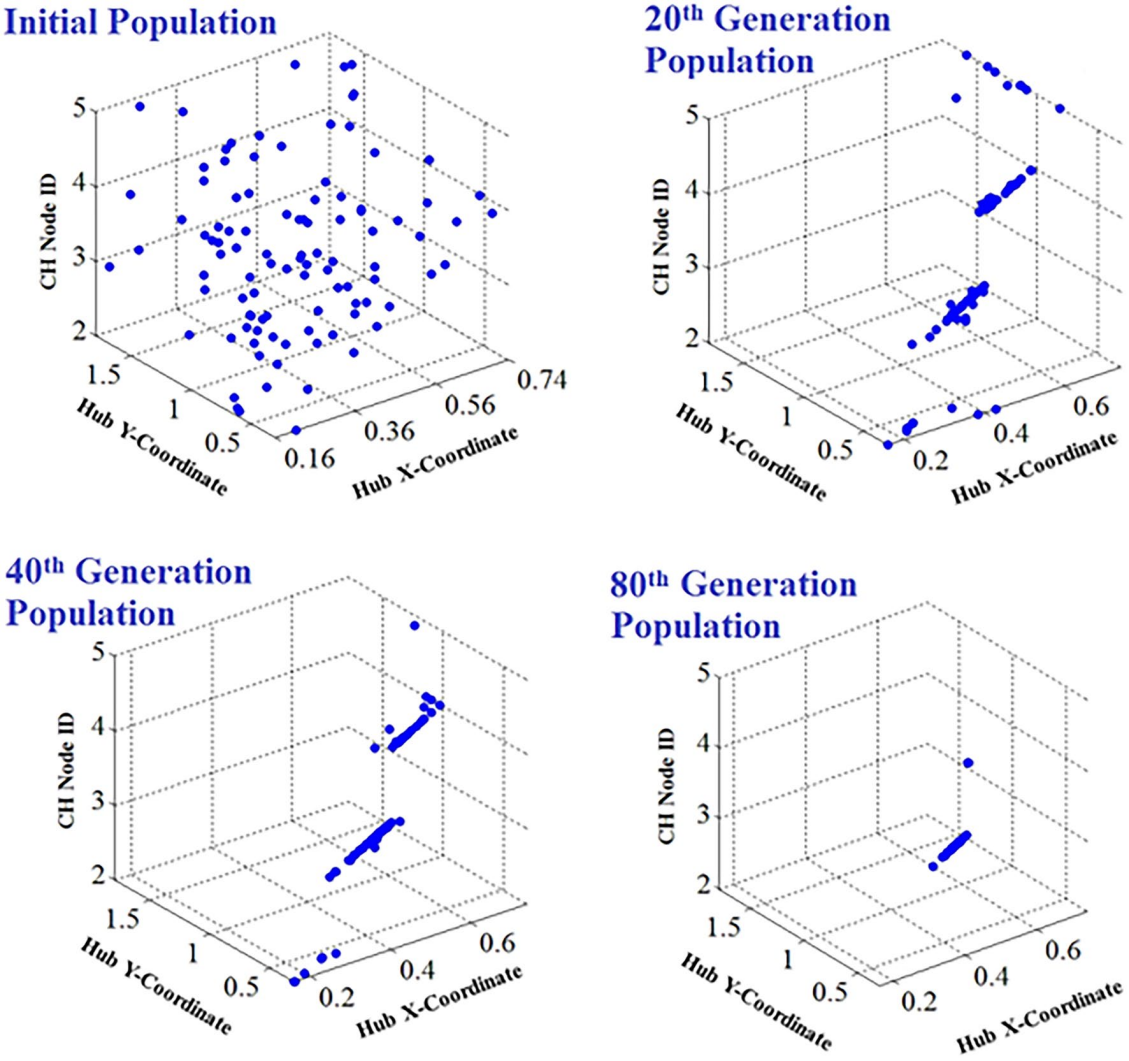
Whale populations for different generations.

**Figure 8:**
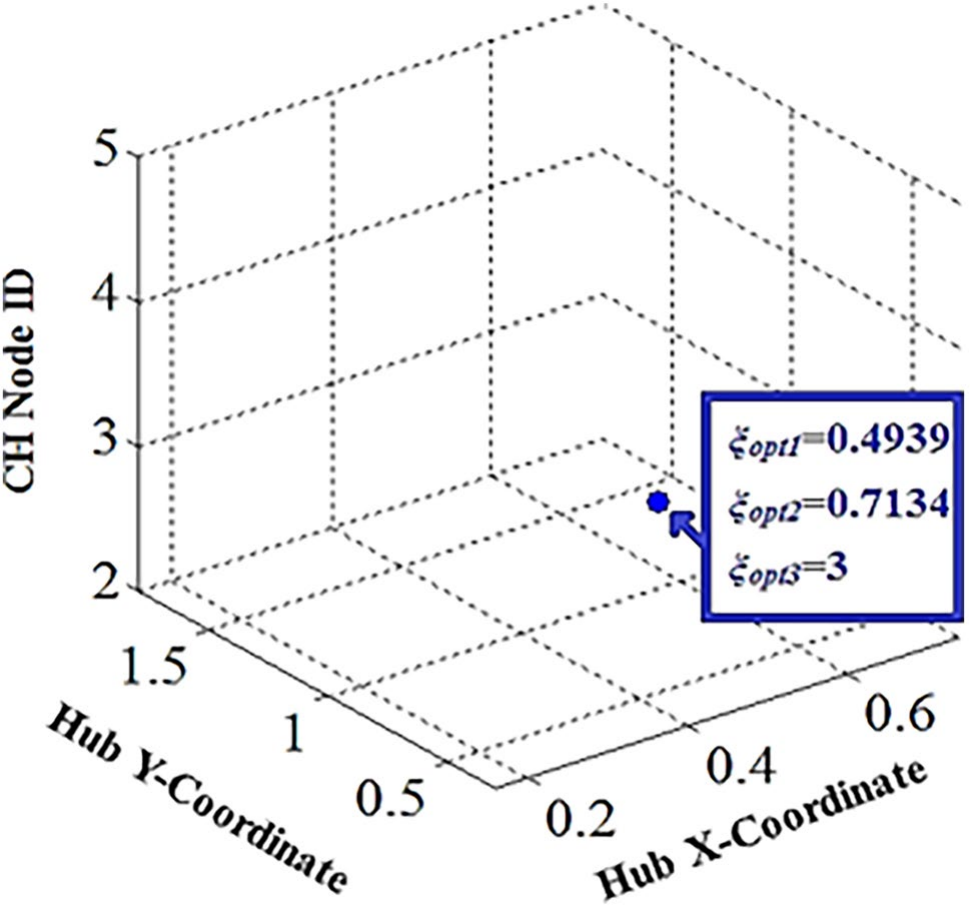
Whale population-final generation.

**Figure 9:**
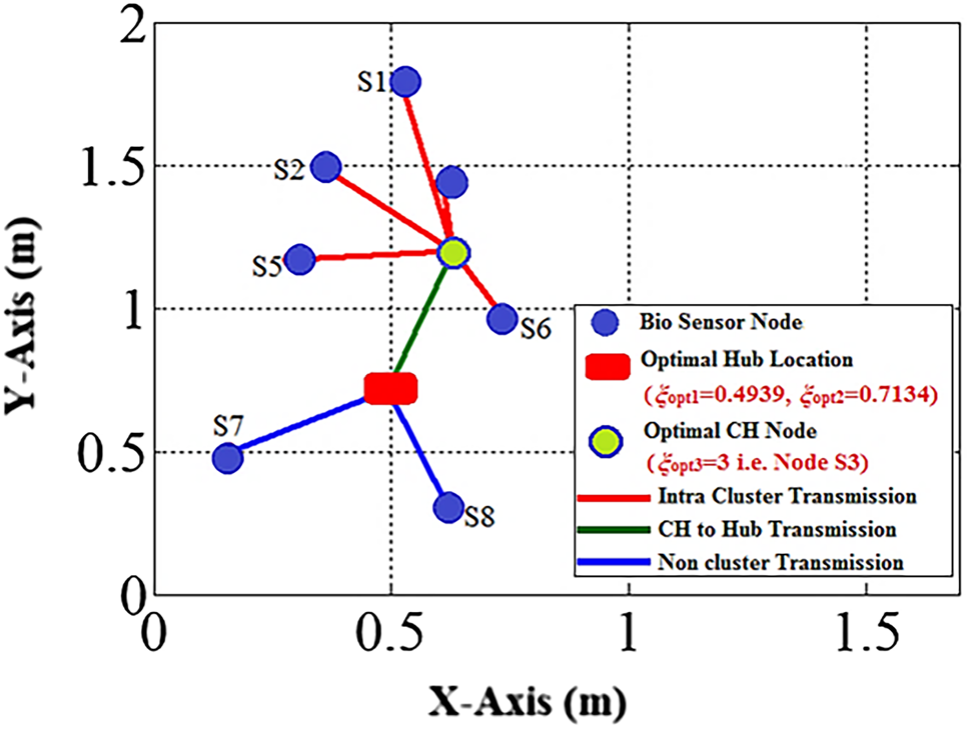
WOA optimal topology of WBAN.

Various generations of the WOA populations are represented by Fig. 7. Figure 8 depicts the WOA’s final generation population. The WOA suggests *ψ*_*opt*_= (*ξ*_*opt1*_=0.4939*, ξ*_*opt2*_=0.7134*,* and *ξ*_*opt3*_=3) as the optimal solution for hub placement. Figure 9 illustrates the recommended optimal topology for the WBAN that was generated by the WOA.

### Complexity and convergence analysis

**Figure 10:**
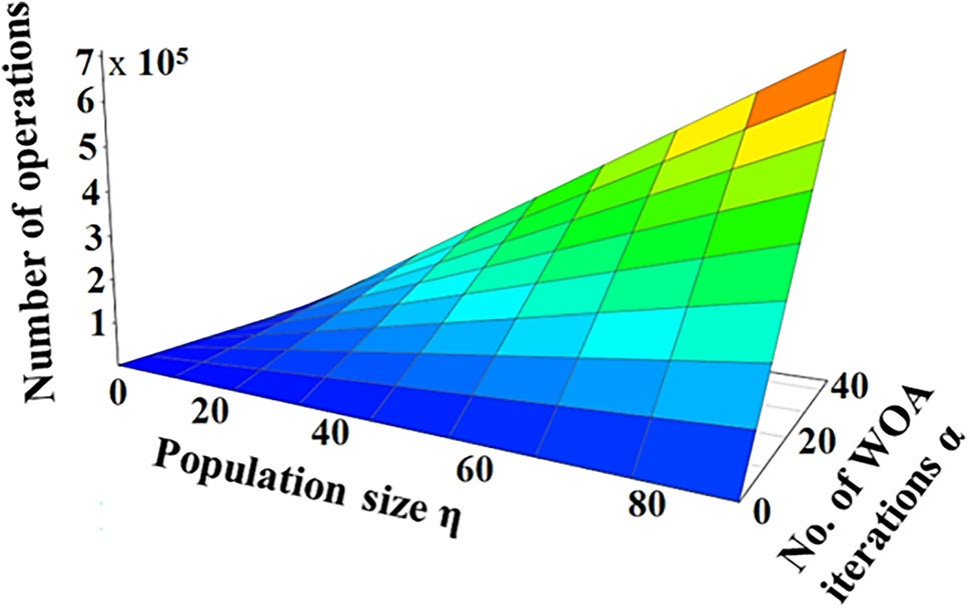
Algorithmic complexity of WHOOP^H^ algorithm.

**Figure 11:**
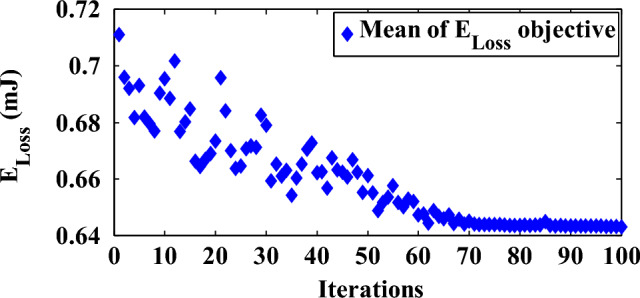
WOA convergence curve.

The computational complexity is based on the number of arithmetical and logical operations required to execute an algorithm. WHOOP^H^ carries out (*180.η.α*+*12.η*+*113*) operations where *η* is the population size and*α* is the iteration count of WOA algorithm. Figure 10 demonstrates how the scalability of *η* and *α* inputs impacts the computational complexity of WHOOP^H^ algorithm. The blue-colored blocks indicate lower complexity levels while the red blocks indicate increased complexity due to high-end inputs. Here the number of WBAN nodes is taken as 8. Hence, the proposed scheme shows the complexity of *O (η.α)* level. The WOA convergence curve of WHOOP^H^ algorithm is shown in Figure 11.

### Data routing

The WOA-based optimum hub node placement technique (WHOOP^H^) is paired with a hierarchical clustering-based energy-efficient routing protocol. This protocol makes use of the WOA’s optimized network topology for the initial round of node transmissions. After the first transmission round, the hub node will continue to be located at the optimal hub position that was suggested by the WOA. However, the CH node will be dynamically re-elected for each subsequent data transmission round. The utilized hierarchical clustering-based routing protocol works in the following phases:

### CPR-MADM based CH selection

The utilized routing scheme makes use of CPR-MADM algorithm for dynamic CH node selection. Here, the CPR ratings are assigned to every cluster node. The CPR rating of a WBAN node is estimated as follows:

26 $${CPR}_{i}=\frac{{E}_{Ri}}{{N}_{CHi}*{D}_{toHub\_i}};\forall i \in U,$$

where ${E}_{Ri}$ signifies the residual energy of node-i;${N}_{CHi}$ signifies that how many times the node-*i* has played the role of CH before the current transmission round; ${D}_{toHub\_i}$ signifies the distance between node-*i* and the hub.

The WBAN node achieving the highest CPR value becomes the CH. Hub node intimates other WBAN nodes regarding the selected CH node by broadcasting a *“CH_SELECT”* packet.

### Data transmission

**Figure 12:**
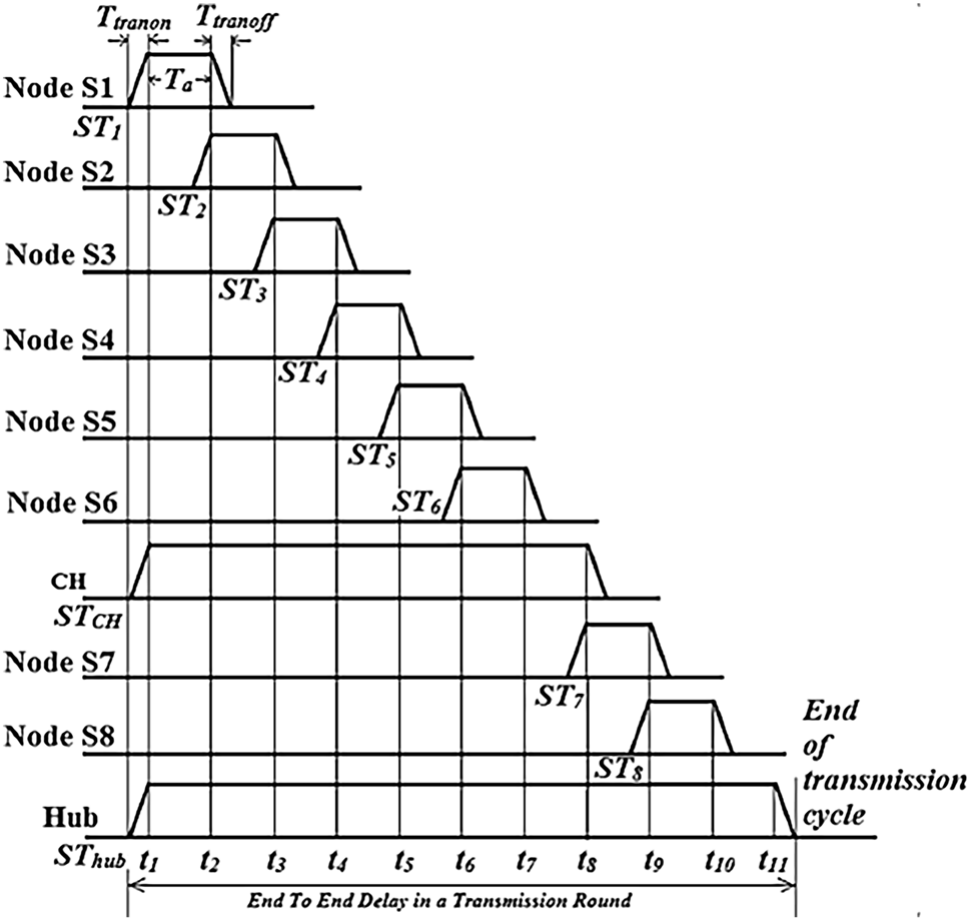
Timing sequence (all nodes are alive).

After CH selection, the physiological parameter data is recorded by the biosensor nodes. Each node in the network receives the TDMA-based duty cycle schedule from the hub. The TDMA schedule is shown in Figure 12. According to this schedule, a network node will stay in standby mode and will become active only during its allotted TDMA slot. The active time period of a node is denoted by the symbol *T*_*a*_. “Standby to Active” and “Active to standby” switching periods are referred to as “*T*_*tranon*_” and “*T*_*tranoff*_”, respectively. The beginning time instances of node-*i*, CH, and Hub are denoted by the letters *STi*, *ST*_*CH*_, and *ST*_*HUB*_, respectively. First, communication occurs between the child nodes and the CH. After then, the CH initiates communication with the Hub. In the end, nodes S7 and S8 that are not part of a cluster communicates to the Hub.

## Protocol simulation

There are different performance parameters such as network stability period, network lifetime, network throughput, and network latency that determine the efficiency of a wireless body area network. In order to simulate these parameters, the WOA-based optimum hub node placement scheme (WHOOP^H^) is applied to the WBAN system shown in Fig. 9. The performance of WHOOP^H^ has been simulated using MATLAB (2008a) tool. Table 3 illustrates the simulation parameters. Furthermore, the performance of the proposed WHOOP^H^ protocol is compared to the state-of-the-art data routing protocols for WBAN systems^4,8,18,19^. The superiority of the suggested protocol is shown via these comparisons.

**Table 3:** Simulation parameters.

Parameter	Value
*E*_*TX*_	16.7E–09 J*/bit*
*E*_*RX*_	36.1E–09 J*/bit*
*E*_*Amp*_	1.97E–09 J*/bit*
*E*_*DA*_	5E–09 J*/bit*
*W*	4000bit
Node’s energy	0.6 J
*d*_*0*_	0.5 m
*F*	2.4e09 *Hz*
*γ, σ, δ*	3.38, 1.5, 0.4 (LOS)
*P*_*t*_	− 5 *dBm*
*P*_*rsens*_	− 101 *dBm*
*T*_*a*_	1 ms
*T*_*tranon*_	0.245 ms
*T*_*tranoff*_	0.25 ms

### Network stability and lifetime periods

Network stability and lifetime periods are indicators of the overall lifespan of a network’s operation. The stability period is the time frame during which all of the nodes of the network are alive and successfully perform all of their duties, such as sensing, processing, and data transmission. The network is considered to be stable during this period of time.

**Figure 13:**
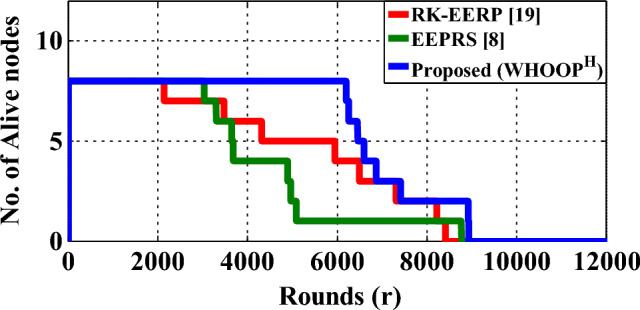
Network lifetime.

The lifetime of the network is defined as the period of time after which all of the network nodes cease carrying out sensing, processing, and data transmission operations. In this work, the stability as well as lifetime periods of a WBAN have been measured by counting the number of transmission cycles that it successfully completed. Figure 13 depicts a comparison of the lifetime curves of different data routing protocols.

According to the findings of the research, the RK-EERP^19^ protocol, the EEPRS^8^ protocol, and the proposed WHOOP^H^ protocol show network stability for 2136, 3037, and 6198data transmission cycles, respectively. Next, the RK-EERP protocol, the EEPRS protocol, and the proposed WHOOP^H^ protocol show the network lifetime of 8407, 8768, and 8937 transmission-rounds, respectively.

The proposed WHOOP^H^ protocol incorporates the WOA-based optimal Hub placement scheme that decides the optimized position for the hub node in WBAN. The optimal hub placement minimizes the amount of total energy that is used by the nodes of the network when they transmit their data packets. Therefore, when compared to the state-of-the-art RK-EERP and EEPRS protocols, the proposed WHOOP^H^ protocol achieves a significant increase in network stability and lifetime periods.

**Figure 14:**
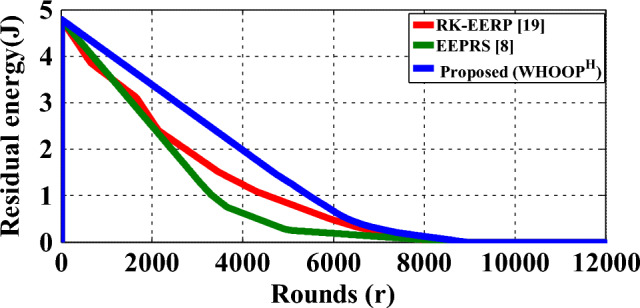
Residual energy plot.

Figure 14 shows the residual energy plots for the proposed WHOOP^H^ protocol, RK-EERP protocol and EEPRS protocol. The residual energy curves of various protocols demonstrate that the suggested WHOOP^H^ protocol provides the slowest rate of energy depletion out of the three protocols. This is the case because the WHOOP^H^ protocol delivers the highest network lifetime. It makes the proposed WHOOP^H^ protocol suitable for use in a WBAN while also improving its energy efficiency.

### Network throughput

**Figure 15:**
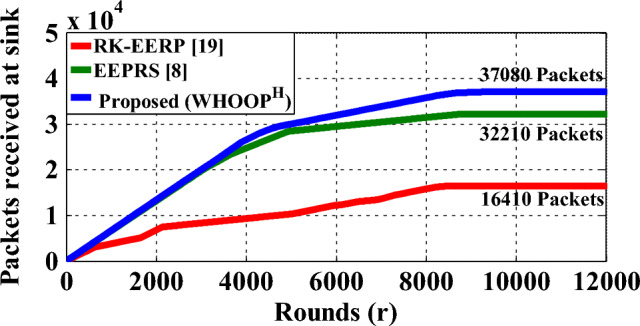
Data packets delivered to hub.

**Figure 16:**
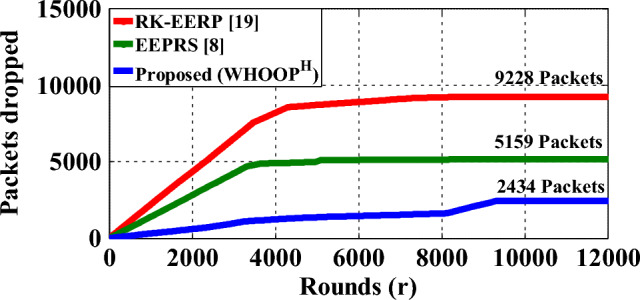
Data packets delivered to hub.

A network's throughput rate is defined as the proportion of data packets that are successfully delivered to the hub within a certain time period. Figure 15 demonstrates the packet count successfully transmitted to the hub, while using the RK-EERP protocol, the EEPRS protocol, and the proposed WHOOP^H^ protocol. Figure 16 shows the count of data packets that were lost while they were being transmitted.

**Figure 17:**
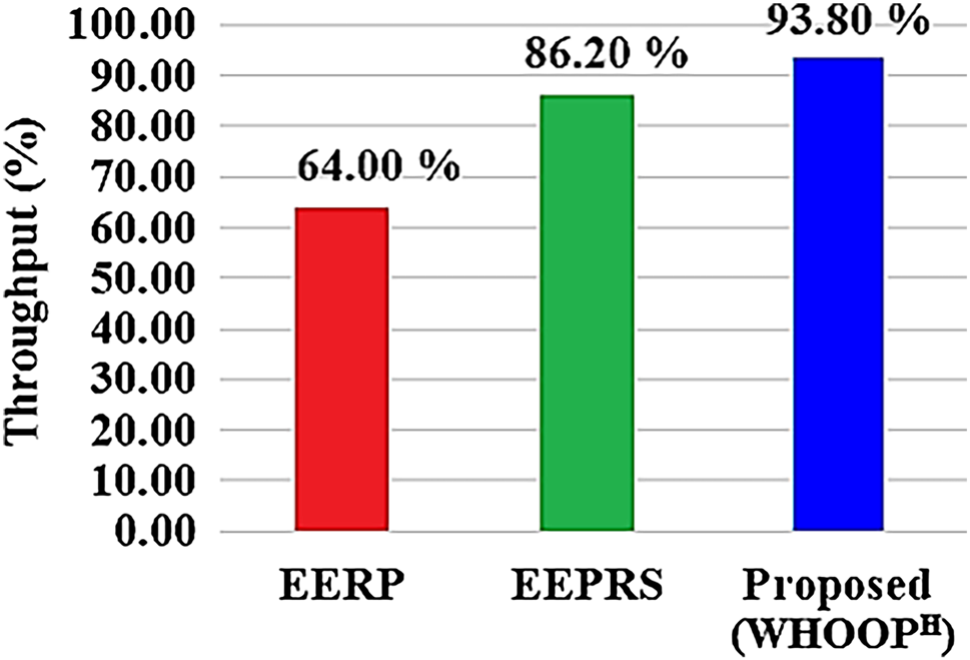
Network throughput.

Based on the findings, it has been observed that the proposed WHOOP^H^ protocol is effective in transmitting 37080 packets to the Hub, whereas 2434 packets are lost in the process of making these transmissions. As a result, the network throughput provided by the WHOOP^H^ protocol reaches as high as 93.8 percent. Figure 17 illustrates the network throughput rates achieved by using different protocols.

### Network latency

**Figure 18:**
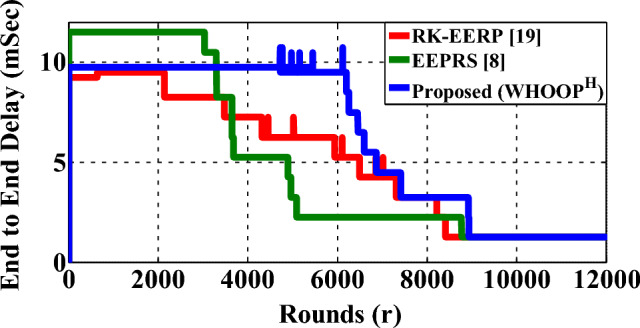
End to end delay (latency).

The end-to-end delay that is seen during the transmission of data packets between the source nodes and the hub is termed network latency. Figure 18 illustrates how different protocols affect the latency of the network. During the stability period of the WBAN, the EEPRS protocol demonstrated a delay of 11.49 msec. EEPRS offers an increased level of delay as it is a multi-hop protocol. The RK-EERP protocol exhibits a latency of 9.29 msec whereas the proposed WHOOP^H^ protocol shows a latency of 9.74 msec. Both the RK-EERP and WHOOP^H^ are clustering-based protocols, and both of them demonstrate lower latency than the multihop EEPRS protocol. Table 4 shows the superiority of WHOOP^H^ protocol by comparing its performance to the various state-of-the-art data routing protocols for WBAN^4,8,18,19^.

**Table 4:** Summary of performance results.

Performance parameter	Stability period	Network lifetime	Throughput	Network latency	Algorithmic complexity
RK-EERP^19^	2136 rounds	8407 rounds	64%	9.29 ms	*O*(*n*^2^)
EEPRS^8^	3037 rounds	8768 rounds	86.20%	11.49 ms	*O*(*n*^2^)
IM-SIMPLE^18^	5300 rounds	7325 rounds	86.3%	11.49 ms	*O*(*n*^2^)
EERP^4^	5315 rounds	6914 rounds	90.31%	12.73 ms	*O*(*n*^2^)
WHOOP^H^	6198 rounds	8937 rounds	93.80%	9.74 ms	*O*(*n*^2^)

## Conclusions and future scope

The lifespan of the wireless body area network operation is significantly impacted by the changes in the position of hub node. As a result, we suggested an optimal hub placement technique (WHOOP^H^) which is based on whale optimization algorithm. The proposed optimal hub placement technique WHOOP^H^ places the hub node at the optimized location within the WBAN. The optimal hub placement minimizes the amount of energy that is used by the nodes of the network when they transmit their data packets.

According to the simulation results, WHOOP^H^ shows the network stability period of 6198 data transmission cycles while the state-of-the-art EERP and the EEPRS protocols show network stability for 2136 and 3037 data transmission cycles, respectively. Similarly, the WHOOP^H^ shows the network lifetime period of 8937 data transmission cycles while the EERP and the EEPRS protocols show network stability for 8407 and 8768 data transmission cycles, respectively. Therefore, when compared to the state-of-the-art relevant protocols, the proposed WHOOP^H^ protocol achieved a momentous growth in network stability and lifetime periods. A network throughput rate of 93.8 percent and a network latency of 9.74 milliseconds are also displayed by the protocol.

In future, we want to develop a WBAN MAC protocol that will allow us to expand our work in case involving multiple WBAN systems. This will prevent problems with interference on different channels.

### Ethical approval

The author (Dr. Shubham Shukla) assures that this article accurately reflects the author's own research and analysis. Co-authors and co-researchers are correctly credited. The findings are correctly positioned in relation to earlier and ongoing studies. Each and every reference is appropriately cited.

## Data Availability

The datasets used and/or analysed during the current study available from the corresponding author on reasonable request.
